# The Challenge for Rapid Detection of High-Structured Circular RNA: Assay of Potato Spindle Tuber Viroid Based on Recombinase Polymerase Amplification and Lateral Flow Tests

**DOI:** 10.3390/plants9101369

**Published:** 2020-10-15

**Authors:** Aleksandr V. Ivanov, Irina V. Shmyglya, Anatoly V. Zherdev, Boris B. Dzantiev, Irina V. Safenkova

**Affiliations:** 1 A.N. Bach Institute of Biochemistry, Research Centre of Biotechnology of the Russian Academy of Sciences, Moscow 119071, Russia; a.ivanov@fbras.ru (A.V.I.); zherdev@inbi.ras.ru (A.V.Z.); safenkova@inbi.ras.ru (I.V.S.); 2 A. G. Lorch Russian Potato Research Center, Kraskovo 140051, Russia; i.shmyglya@mail.ru

**Keywords:** isothermal amplification, lateral flow assay, potato spindle tuber viroid, recombinase polymerase amplification

## Abstract

An assay was developed to detect the potato spindle tuber viroid (PSTVd), a dangerous plant pathogen that causes crop damage resulting in economic losses in the potato agriculture sector. The assay was based on the reverse transcription and recombinase polymerase amplification (RT-RPA) of PSTVd RNA coupled with amplicon detection via lateral flow assay (LFA). Primers labeled with fluorescein and biotin were designed for RT-RPA for effective recognition of the loop regions in the high-structured circular RNA of PSTVd. The labeled DNA amplicon was detected using lateral flow test strips consisting of a conjugate of gold nanoparticles with antibodies specific to fluorescein and streptavidin in the test zone. The RT-RPA-LFA detected 10^6^ copies of in vitro transcribed PSTVd RNA in reaction or up to 1:10^7^ diluted extracts of infected plant leaves. The assay took 30 min, including the RT-RPA stage and the LFA stage. The testing of healthy and infected potato samples showed full concordance between the developed RT-RPA-LFA and quantitative reverse transcription polymerase chain reaction (RT-qPCR) and the commercial kit. The obtained results proved the feasibility of using the developed assay to detect PSTVd from a natural source.

## 1. Introduction

The potato spindle tuber viroid (PSTVd) is the most primitive potato plant pathogen that causes perceptible crop damage [1]. PSTVd can infect different hosts belonging to the Solanaceae family. As a part of the Pospiviroidae family, PSTVd is a circular, rod-like, structured RNA molecule with a central conservative region and variable “rod” terminals. There are more than 200 PSTVd variants with an average length of 359 nt. The secondary structure of PSTVd is non-complete double stranded RNA (dsRNA), consisting of loops and non-canonical base-pairing [2]. The RNA molecules can be divided into five domains: terminal right, terminal left, central conservative regions, pathogenicity-related P domains, and variable V domains [3]. PSTVd is metastable RNA—the domains can be altered under some physiological conditions or due to their interactions with proteins. Under physiological conditions, the rod-like structure prevails [2]. The viroids neither contain a protein capsid nor encode any protein. Pospiviroidae contain special features in the sequence and structure of their conservative region that allow for the hijacking of host cell machinery for asymmetric rolling-circle replication of the viroid [4,5], trafficking among nucleolus and other cellular compartments [6], and intercellular transport through plasmodesma [7,8]. It is still under debate (see review [9]) whether the exact mechanism of pathogeny occurs through regulation of the protein phosphorylation status, as a source of short RNA causing mRNA degradation [10], or through changing methylation of genomic DNA [11].

PSTVd can spread throughout hosts vertically—through seeds and vegetative propagation—and horizontally through pollen. The infected pollen can transmit the viroids to the same host species as well as other host species [12]. When potato plants are infected by severe strains of PSTVd, they demonstrate specific symptoms: smaller leaves, spindle-shaped tubers, knob-like protuberances, or multiple small tubers. However, mild strains of PSTVd often cause no symptoms. Tuber shrinking as a result of the infection causes a potato yield reduction of up to 64% [13].

The infection cannot be treated, so a PSTVd diagnostic is the only way to stop it from spreading and decrease economic losses. The nature of PSTVd restricts its detection based on antigen–antibody recognition. To date, no antibodies against the viroid have been found. Only nucleic acid detection methods can be applied, with the first methodological group being the hybridization of PSTVd with labeled oligonucleotides [14,15,16,17,18]. These methods are time-consuming, qualitative or semi-quantitative, and less sensitive than modern approaches. Currently, these methods are rarely used. Today, the main method for detecting PSTVd is reverse transcription polymerase chain reaction (RT-PCR). Qualitative tests of PSTVd with RT-PCR followed by electrophoresis have been proposed [17,19,20,21,22]. However, the quantitative RT-PCR (RT-qPCR) method for PSTVd detection is most commonly used. Most research studies do not give exact detection limits for their assays; they only report the sensitivity of the RT-qPCR in serial diluted extracts of infected plant leaves: 1:10^6^ [23] and 1:10^4^ [24,25]. The European and Mediterranean Plant Protection Organization (EPPO) recommended conducting some of these tests for highly sensitive PSTVd detection [26]. While the official standards of the EPPO state that the dilution rate is the limit of detection (LOD) [27], only one LOD was found: 17 pg of PSTVd RNA (8.4·10^7^ copies) for infected samples after specific RNA extraction [28]. RT-qPCR requires the use of expensive equipment, which may be an obstacle for introducing the method into the field as a detection practice.

An alternative way of detecting nucleic acid is isothermal amplification. This method does not require complex or expensive equipment. Thus, one isothermal test, nucleic acid sequence-based amplification (NASBA), was developed for detecting PSTVd; it has a sensitivity equal to 250–2500 copies of the RNA [29]. Another isothermal point-of-care test, the loop-mediated isothermal amplification (LAMP) test, detected 10^3^ dilution of PSTVd (100–1000 copies in the sample) and was verified by a large-scale field trial of more than 400 samples [30]. The LAMP-based assay, suggested by Lenarčič et al., detected PSTVd in 10^4^ times diluted potato samples [31]. Another study designed a prototype of the LAMP-based test for PSTVd [32]. The test was able to detect 10^4^ copies of the PSTVd DNA template. Different isothermal tests have been developed for other viroids. Isothermal and chimeric primer-initiated amplification of nucleic acids was used to test for the chrysanthemum chlorotic mottle viroid [33]. One possible application of the isothermal amplification method is recombinase polymerase amplification (RPA) [34]. RPA proceeds for 20 min at 37–42 °C, and it can be coupled with real-time detection or point-of-care detection with a lateral flow assay (LFA). The only known RPA-LFA-based test for a viroid detects tomato chlorotic dwarf viroid (TCDVd) up to 100 fg–1 pg (5·10^5^–5·10^6^ copies) of pure in vitro transcribed RNA [35].

For the research study discussed in this paper, the first RPA-LFA for detecting PSTVd was developed. This study included selecting optimal primers for effective recognition of the loop regions in high-structured circular RNA. The presented comparison demonstrates tools for further work on peculiar analytes, such as viroids.

## 2. Results and Discussion

### 2.1. Selection of Primers for Recombinase Polymerase Amplification Lateral Flow Assay (RPA-LFA)

The linear PSTVd RNA was synthesized with in vitro transcription and obtained as a uniform product of 357 nt according to gel-electrophoresis (data presented in Supplementary Material, Figure S1). The absence of DNA in the RNA preparation was confirmed via qPCR fluorescence curves and melting peaks specific to the full-length DNA PSTVd product (Supplementary Material, Figure S2). According to Gast et al., the structure and conformation properties of in vitro transcribed PSTVd and native PSTVd are similar [4]. 

The first stage of developing the RPA-LFA for PSTVd involved designing a set of labeled primers and testing them with model PSTVd RNA. Three primers were designed, labeled by FAM and five primers labeled by biotin (Table 1). 

**Figure 1:**
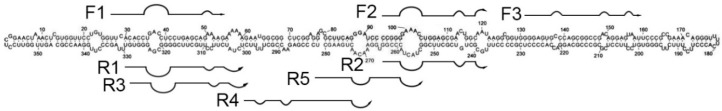
Scheme of hybridization of the designed primers with a PSTVd RNA molecule. Data about the structure of PSTVd were obtained from Botermans et al. [24] and combined with the location of the studied primers.

The primers were aligned along the PSTVd molecule to locate the 3’ end of the primer near the unpaired loop regions of PSTVd RNA (Figure 1). This was done to facilitate hybridization of the DNA primers with high-structured PSTVd RNA at the temperature of the RT-RPA (39 °C). A similar approach was used in other studies to design PCR primers [17,24] and NASBA [29] for PSTVd detection, where the loops that were chosen were the same as those for the annealing site of the primers.

**Figure 2:**
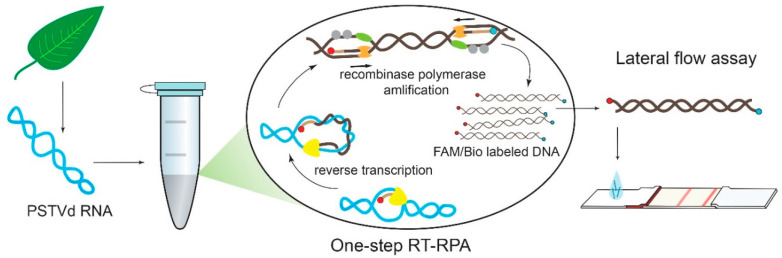
Scheme for PSTVd detection via RPA coupled with detection using a lateral flow test strip.

The obtained combinations of the primers were tested by RT-RPA-LFA. The lengths of the designed corresponding amplicons were similar, and ranged from 135 bp to 303 bp (see Supplementary Material, Table S1), which is an appropriate length for LFA detection according to our previous results [36]. The scheme for the RPA coupled with detection using a lateral flow test strip is presented in Figure 2.

**Figure 3:**
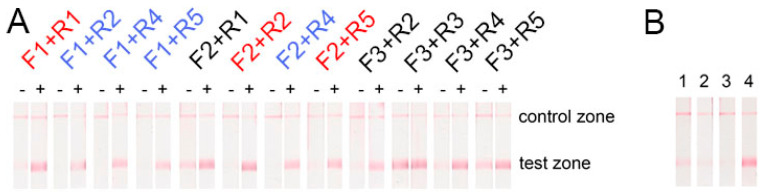
Test of the false-positive signal of LFA after using different primers in: (**A**) reverse transcription (RT)-RPA; “-” means no RNA was added, “+” means 10^10^ copies of PSTVd RNA were added in the RPA reaction. The primer pairs that were expected to form dimers are depicted in red, pairs that demonstrated no non-specific signal are depicted in blue, and pairs that formed an unpredictable non-specific signal are depicted in black; (**B**) RPA without Moloney murine leukemia virus (MMLV); (1) F1 + R2, (2) F1 + R4, (3) F1 + R5, (4) F2 + R4.

RPA-LFA was performed for two cases: (1) with 10^10^ copies of PSTVd RNA and (2) without the RNA template to register the sticking primers and the non-specific signals. Strong positive signals (bright red lines in the test zones, Figure 3A) were obtained for all the primers. Primers with partial complementarity (F1-R1, F2-R2, F2-R5; see Table 1, Figure 1) demonstrated the formation of cross dimers, leading to weak false-positive signals (Figure 3A). Moreover, pairs with F3 primers also led to false-positive signals. Consequently, several combinations of primers without non-specific signals in the test zone were found (see Figure 3A): F1 + R2, F1 + R4, F1 + R5, and F2 + R4.

The next selection stage for the five chosen primer pairs was performed using RPA-LFA with 10^10^ copies of the target RNA, but without Moloney murine leukemia virus (MMLV) reverse transcriptase. This stage had RNA-guided non-specific effects, which can be detected upon a high concentration of total RNA in an analyzed sample. Notably, a false-positive signal in four pairs of the chosen primers was observed. Only the F1 + R5 combination demonstrated no visible signal in the test zone of the test strips (Figure 3B). Thus, this proved the absence of the template DNA before the RPA experiments by qPCR (see Supplementary Materials, Figure S1). Perhaps PSTVd RNA induces either unspecific binding of both primers or their unspecific binding with components of the reaction. For subsequent manipulation, primers F1 and R5 were used. The F1 binding site belongs to the P domain of PSTVd, and the R5 binding site belongs to the central domain of PSTVd.

The feasibility of using the F1 and R5 primers to detect different strains of PSTVd was estimated bioinformatically. All full-genomic 349 sequences of the PSTVd isolates presented at the GeneBank were analyzed (see Figure S3 of Supplementary and Files S1 and S2). A total of 167 sequences (47.8%) were found that contain primer-binding sites that are fully complementary to the F1 and R5 primers that were studied. The selected primers cover almost half of all known PSTVd isolates. In all, 61% and 79% of the sequences contain the sites that are full complementary to the F1 and R5 primers, respectively. Moreover, it can be expected that the number of detected isolates will be higher. This is evidenced by Boonham et al. [23], which showed the successful recognition of some PSTVd isolates with a mismatch at the annealing site using RT-PCR.

### 2.2. Characterization of Reverse Transcription (RT)-RPA-LFA

**Figure 4:**
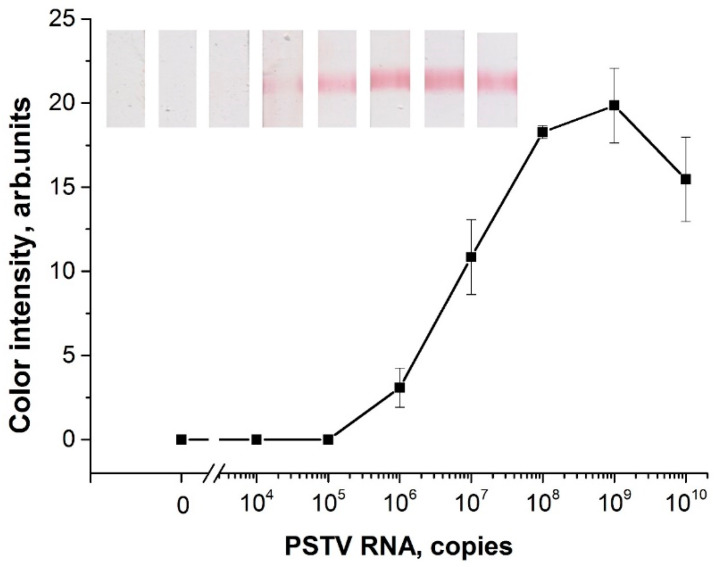
Dependence of color intensity in the test zone after RT-RPA-LFA from an initial amount of transcribed PSTVd RNA in a test tube. The scans of the strips correspond to the plotted values.

First, the LOD of RT-RPA-LFA was determined using samples of serial diluted linear PSTVd RNA (in water) from 10^10^ to 10^4^ copies. The RT-RPA-LFA based on the selected primers (F1, R5) was able to detect 10^6^ copies of PSTVd RNA in a tube (Figure 4). This result coincides with the RT-RPA-LFA results for TCDVd detection with an LOD that equaled 5·10^5^–5·10^6^ copies of in vitro transcribed RNA [35]. Moreover, the obtained LOD of PSTVd RNA was lower than the LOD (8.4·10^7^ copies) previously demonstrated by PCR [22,27]. The RT-RPA-LFA was carried out for 30 min at 37 °C, with 20 min for the RT-RPA stage and 10 min for the LFA stage. RT-RPA-PCR did not require a thermal cycler, and it was faster than the RT-PCR of PSTVd described.

**Figure 5:**
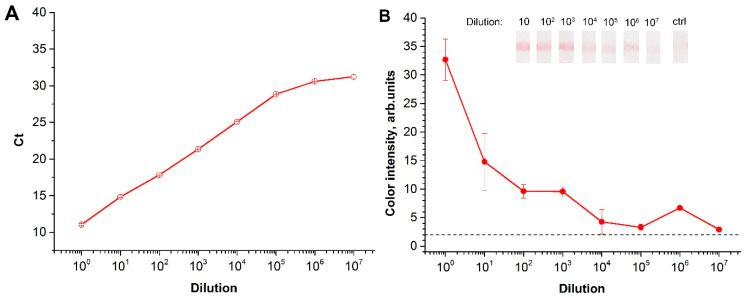
Analysis of the extracts of infected plant leaves: (**A**) dependence of Ct after quantitative reverse transcription polymerase chain reaction (RT-qPCR) for different dilutions of the infected sample. (**B**) Dependence of color intensity in the test zone after RT-RPA-LFA from a dilution of the infected sample. “Ctrl” refers to the control strip after LFA of RNA extraction from a non-infected plant.

For the natural viroid, the sensitivity of the RT-RPA-LFA was estimated using serial dilutions of the leaf homogenates of infected in vitro potato cultures. For two of the infected in vitro potato cultures, the initial leaf homogenates were diluted in the homogenate of healthy plants. The total RNA was then extracted. First, the presence of PSTVd RNA was confirmed via RT-PCR. The serial-diluted extracts were also evaluated. The RT-qPCR of the samples showed sensitivity equal to 10^7^ fold dilution (Figure 5A). The RT-RPA-LFA was able to detect PSTVd in the samples that were diluted up to 10^7^ (Figure 5B). However, the developed RT-RPA-LFA demonstrated greater variation between repetitions, and it could not be approximated for quantitative calculations comparing commercial RT-qPCR. Therefore, the LOD obtained was slightly inferior to the commercial RT-PCR, but better to what was previously presented 1:10^4^ [24,25] and 1:10^6^ [23]. Moreover, EPPO official standards approved a 1:10^6^ dilution for diagnosing the viroid [27]. 

### 2.3. RT-RPA-LFA of Healthy and Potato Spindle Tuber Viroid (PSTVd)-Infected Potatoes 

**Figure 6:**
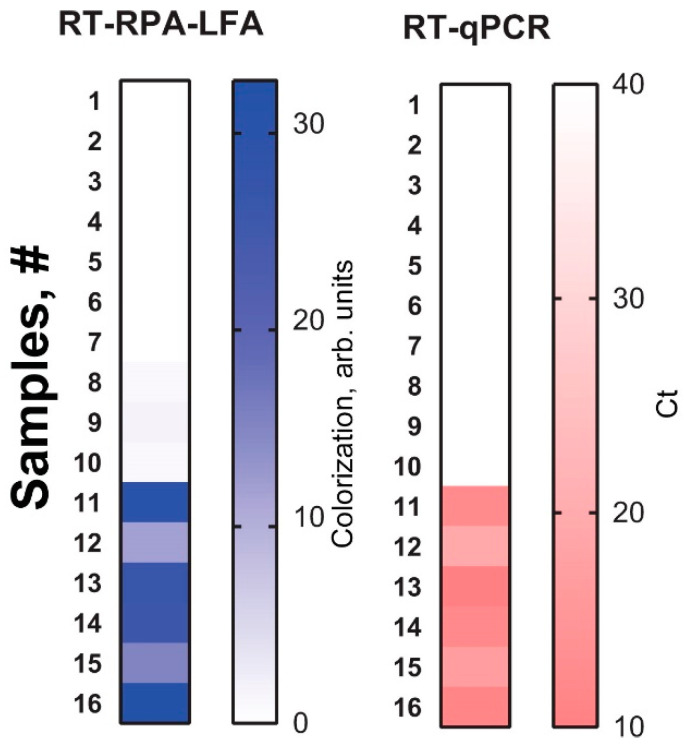
Detection of PSTVd in potato leaves by RT-RPA-LFA and RT-qPCR. The results for each sample are presented as distinct squares in the heat maps.

The potato samples were tested using healthy in vitro plants (*n* = 10) and infected in vitro plants (*n* = 6). The results were interpreted as positive if the means of the duplicated RT-RPA-LFA were above 2 arbitrary units. The RT-RPA-LFA showed 10 negative (healthy) and 6 positive (infected) samples. The results were fully confirmed using the commercial RT-qPCR kit. Figure 6 demonstrates the results obtained as squares. The more intense the shade, the more positive the sample. The heat maps for both RT-RPA-LFA and RT-qPCR visually represent the overlapping results of the two methods. Thus, the test proved the feasibility of using the developed assay to detect PSTVd from a natural source. 

## 3. Materials and Methods

### 3.1. In Vitro Transcription of PSTVd RNA

A sequence containing the PSTVd (Russian isolate, GenBank accession EU257478) gene flanked with the T7 promoter and the T7 terminator was synthesized and cloned in a pUC19 vector by Evrogen (Russia). A plasmid pUC19-T7-PSTVd was linearized by the *HindIII* enzyme (NEB, USA). The PCR product of the gene was used as a DNA template for in vitro transcription. PCR was performed using M13 reverse (AGCGGATAACAATTTCACACAGGA) and PSTV reverse (AGGAACCAACTGCGGTTCC) primers and a Q5^®^ High-Fidelity DNA polymerase PCR mix (NEB). The primers were synthesized by Evrogen. The DNA fragment contained the T7 promoter, PSTVd gene, and T7 terminator. The PCR products were purified from a PCR mix with a Monarch^®^ DNA Extraction kit (NEB, USA). The DNA fragment was added to up to 50 ng/µL (180 nM) of reaction mix containing 166 mM HEPES-KOH pH 7.5, 32 mM MgCl_2_, 40 mM dithiothreitol, 2 mM spermidine, 100 mg/mL bovine serum albumin (BSA), 7 mM each nucleoside triphosphate (NEB), 2 U/µL RiboLock (NEB), and 5 U/µL T7 polymerase (NEB). The transcription was carried out for 2 h at 37 °C.

### 3.2. Purification of PSTVd RNA

Extraction of PSTVd from the reaction mix was performed as described [33,37] with some modifications. The transcription mix was treated with acidic phenol-chloroform solution, mixed, and then centrifuged (10,000× *g*). The RNA was selectively precipitated by the addition of LiCl to the top fraction up to 3M, and incubated for 30 min at 4 °C. The pellet was diluted in deionized water and desalted using the gel-filtration spin column Zeba Spin MWCO 7K (ThermoScientific, Waltham, MA, USA). To remove minor residue of the DNA template, the solution was treated with DNAseI (NEB), as recommended in the manual. Finally, the RNA was precipitated with 70% ethanol and 85 mM sodium acetate. The RNA pellet was washed with 70% ethanol and dissolved in deionized diethylpyrocarbonate-treated water. The integrity of the PSTVd RNA was estimated using electrophoresis in polyacrylamide gel. The absence of DNA in the RNA solution was verified by RT-qPCR. Concentrations (ng/µL) of the RNA were automatically measured by NanoDrop2000 (ThermoScientific, USA) based on the Beer–Lambert equation and characteristic extinction coefficient for RNA (40 ng-cm/µL). The molar concentration and the number of copies were calculated for the length of the transcript equal to 357 nt, the results are presented in Supplementary Material, Section 1.

### 3.3. Preparation of Conjugate of Gold Nanoparticles with Antibodies

Conjugate of gold nanoparticles (GNP) with mouse monoclonal antibodies specific to fluorescein (anti-FAM) (clone 2A3c, Bialexa, Russia) and LFA test strips were prepared as described at [38].

Briefly, to synthesize the GNP with a diameter of 17 nm, 1 mL of 1% HAuCl_4_ and 95 mL of deionized water were mixed and heated to boiling point, then 4 mL of 1% sodium citrate was added and boiled for 25 min. For the synthesis of the GNP–anti-FAM conjugate, anti-FAM and GNP were adjusted to pH 9.0, mixed with a final ratio of 10 µg of anti-FAM per 1 mL of GNP solution with optical density at 520 nm (OD_520_) = 1.0, incubated for 1 h, blocked by BSA (Sigma, St. Louis, MO, USA) to 0.25%, and separated from the unbinding proteins via centrifugation at 10,000× *g* for 30 min at 4 °C.

### 3.4. Preparation of LFA Test Strips

The GNP–anti-FAM conjugate (OD_520_ = 4.0) was dispensed at 3.2 μL per 1 mm of strip width on a PT R5 fiberglass membrane (Advanced Microdevices, Santa Clara, CA, USA). Streptavidin (Imtech, Russia; 1 mg/mL) and goat anti-mouse IgG (Imtech; 1 mg/mL) were dispensed at 0.15 μL per 1 mm of strip width on test and control zones of nitrocellulose membrane CNPC12 (Advanced Microdevices), respectively, using an IsoFlow Dispenser (Imagene Technology, Lebanon, NH, USA). Test strips were assembled using the sample membrane GFB-R4, final adsorbing membrane AP045 (Advanced Microdevices), and the aforementioned fiberglass and nitrocellulose membranes. The multimembrane composites were cut and packed according to Byzova et al. [38].

### 3.5. Sample Collection and Characterization 

Healthy (*n* = 10) potato plants and potato plants artificially infected by PSTVd (*n* = 6) were grown in vitro and provided by I.V. Shmyglya (A.G. Lorch Russian Potato Research Center, Kraskovo, Moscow region, Russia). The infection did not demonstrate any visible symptoms. Samples of potato leaves (150 mg) were homogenized in a Petri dish. The extraction of total RNA from the samples was performed using a commercial total plant RNA extraction kit (Syntol, Russia) according to the manufacturer’s protocols. The extracts were tested using a commercial qPCR kit (Syntol) prior to the subsequent applications.

### 3.6. Primer Design for RPA

In general, primers for RPA were designed according to the recommendations of TwisDx (UK). However, the 3′ of the primers were designed more specifically to hybridize with loop regions of PSTVd. The prediction of primer dimerization and properties were obtained using OligoCalc [39] and ThermoFisher’s Multiple Primer Analyzer (ThermoFisher, Waltham, MA, USA) online software. Table 1 illustrates the primer sequences.

The specificity of the chosen primers pair among the different PSTVd strains and isolates was estimated by multiple alignments of all 349 of the GenBank-deposited full genome sequences of the PSTVd isolates. The analysis was performed using Jalview software [40].

### 3.7. RPA with the LFA test

An RPA TwistDx kit (TwistDx, UK) was utilized for the RPA according to the manufacturer’s protocols with modifications [41]. Briefly, 300 nM FAM and biotin labeled primers (Syntol) were added to the rehydration buffer, as well as 300U MMLV reverse transcriptase (Evrogen), 0.2 U/µL RNAse inhibitor (Thermo Fisher), followed by 10 µL of samples with a template. The PSTVd RNA or analyzed sample was used as a template. A lyophilized pellet from the kit was dissolved in the mix. To start the reaction, 14 mM magnesium acetate was added. RPA was carried out at 39 °C for 20 min using BioRad T100 Thermal Cycler (USA). After the reaction, 5 µL of the RPA mix solution was added to 65 µL of PBS and used as a sample for LFA. The test strip was submerged in the tested sample for 2 min at 37 °C. The qualitative results (with colorization of the test zone indicating the presence of PSTVd in a sample) were estimated visually after 10 min, and the visual LOD of the LFA was defined as the concentration of a target when the test line appeared. To quantify the results, the test strips were scanned using a Canon 9000F Mark II scanner (Canon, Tokyo, Japan), and the digital images were analyzed with a TotalLab TL120 (Nonlinear Dynamics, UK). Approximations for the initial sections of the dependence of the color intensity of the LFIA test line on the viroid’s concentration were constructed using Origin Pro 9.0 (OriginLab, Northampton, MA, USA). The measured value of 2 arbitrary units accorded to the appearance of the visible test zone. For the calibration curve, each point was measured in duplicate.

## 4. Conclusions

The PSTVd is a highly infectious viroid that causes severe damage to crops. Rapid and sensitive detection methods are essential to prevent viroid spread. We developed a rapid (30 min) RT-RPA-LFA for detecting PSTVd. The main advantage of the RT-RPA assay is the ability to carry out all the reactions at the same temperature (39 °C). RT-RPA, in contrast to the most widespread RT-PCR technique, does not require cyclic temperature changes. This simplifies the instrument base for the analysis and provides the possibility of widespread monitoring of the viroid, even in field conditions. Such a tool is especially important due to the lack of immune tests for viroids, which serve as primary screening tools for other phytopathogens.

Through a design of loop-targeting primers and their screening, we overcame the limitation of isothermal amplification of high-structured circular RNA. Consequently, an RT-RPA-LFA with a LOD equal to 10^6^ copies of PSTVd RNA in a reaction of up to 1:10^7^ diluted plant samples. This sensitivity is close to the previously reported RT-PCR for PSTVd. The simplicity and sensitivity of the developed assay indicated above determine its competitive potential for on-site control and efficient crop protection.

## Figures and Tables

**Table 1:** Primers for recombinase polymerase amplification lateral flow assay (RPA-LFA).

Name	5′-3′ Sequence	Position at RNA of Potato Spindle Tuber Viroid (PSTVd)	Modification
PSTV F1 ^1^	ggttcacacctgacctcctgagcagaaaag	25–54	FAM
PSTV F2	cggggaaacctggagcgaactggcaat	94–120	FAM
PSTV F3	gggagtgcccagcggccgacaggagtaatt	131–160	FAM
PSTV R1	accctcgccccgaagcaagtaagatag	301–327	Biotin
PSTV R2	accgggtagtagccgaagcgacagcgc	239–265	Biotin
PSTV R3	caccctcgccccgaagcaagtaagatagag	299–328	Biotin
PSTV R4	aaaaagcggttctcgggagcttcagttgtt	269–298	Biotin
PSTV R5	ggagcttcagttgtttccaccgggtagtag	254–283	Biotin

^1^ F: a forward primer, R: a reverse primer.

## Supplementary Materials

The following are available online at https://www.mdpi.com/2223-7747/9/10/1369/s1, Figure S1: Gel-electrophoresis of PCR fragment of PSTVd DNA and PSTVd RNA after transcription, Figure S2: DNA contamination test after purification of in vitro transcribed PSTVd RNA, Figure S3: List of the 349 PSTVd full-genome sequences deposited at GeneBank with highlighted primers binding sites. Table S1: Lengths of the amplicons. File S1: List of the full-genomic sequences of PSTVd strains. File S2: List of the PSTVd sequences containing both F1 and R5 primers binding sites.
